# Preparation and storage stability of meat spread developed from spent hens

**DOI:** 10.14202/vetworld.2015.651-655

**Published:** 2015-05-23

**Authors:** Ashish Kumar, S. K. Mendiratta, Arup Ratan Sen, G. Kandeepan, Suman Talukder, Heena Sharma, Arvind Soni, A. Irshad, Sanjay Kumar

**Affiliations:** 1 Division of Livestock Products Technology, Indian Veterinary Research Institute, Izatnagar, Bareilly - 243 122, Uttar Pradesh, India; 2 National Research Centre on Meat, Hyderabad - 500 092, Telangana, India

**Keywords:** meat spread, microbiological quality, sensory scores, spent hens, storage stability, thiobarbituric acid reactive substances, total plate count

## Abstract

**Aim:**

The present study was carried out to develop a meat spread as a healthier alternative to already existing meat products utilizing undervalued spent hen meat to add a new dimension to meat products.

**Materials and Methods:**

Carcasses were processed within 30 min of slaughter and conditioned at 4±1°C for about 24 h and then braised along with other ingredients to get the final product. The products were evaluated for proximate composition, peroxide values, pH, microbiological, and sensory qualities as per standard procedures.

**Results:**

The mean percent values for moisture, crude protein, ether extract, and total ash content of developed product were 58.75±0.32, 9.12±0.44, 11.19±0.16, and 2.35±0.17, respectively. No significant difference was observed for mean coliform and the yeast and mold counts with the progression of storage period, but samples differed significantly for mean pH, thiobarbituric acid and total viable plate count during storage of meat spread. A progressive decline in mean sensory scores was recorded along with the increase in storage time.

**Conclusion:**

The meat spread was found to be a good alternative to process the underutilized spent hens for its efficient utilization for product development.

## Introduction

India ranks third in the egg production and fifth in the broiler production [1,2]. Total chicken and layer population of India for the year 2012 were 974 million and 310 million, respectively [3]. As per Food and Agricultural Organization projections, global per capita meat requirement will increase above 20% between 2006–2008 and 2050 due to emerging economies such as China and India [4]. However, the meat products which were considered as the most healthy food component of the diet are surrounded by controversies, which may be due to associated excessive calories leading to obesity, high cholestremia or the complex processing techniques including wide range of food additives in the food articles. These are only some of the factors, which are leading the list associated with the increasing concerns associated with the consumption of meat products.

Hence, in the current scenario as per the changing demand of the society we need the following objectives to be achieved while developing new meat products or upgrading the existing ones, i.e., first, it should be developed from the naturally occurring raw materials; second, it can be consumed as a part of daily life; third, the meat product developed should have undergone minimum processing including the loads of additives it is carrying and lastly it should be economical and involving minimum impact to the environment. Today, the meat from layers is usually considered a by-product of the egg industry and usually fetches lower market prices as compared to broiler meat. Globally, spent hens were used not for human consumption, but there are about 2.6 billion used in the pet food industry [5]. Spent hen meat is a good source of nutrients such as proteins and omega-3 fatty acids. However, the meat is very tough and chewy [6]. Spent hens have an important place in Indian culinary practices for human consumption after the end of economic laying cycle [7]. In the era of increased demand, we cannot afford to underutilize such a valuable source of animal protein. Spent hen meat is more suitable for processing to value added/convenience products [8] and the undesirable characteristics of these meats may not be reflected in final products due to non-meat ingredients [9]. Many such products like cheese spread, mayonnaise, jam, jelly are present in the market. As far as India is concerned, no spreadable meat product is marketed yet. Thus, the spreadable meat product will add a new dimension to convenience food and poultry meat may be a better option for the preparation of spread.

Efficient utilization of spent hen meat in foods may revolutionize meat industry by standardizing appropriate and economic technology for processing such underutilized meat into value-added meat products that are palatable and economically viable [10]. Spreadable products are a kind of convenience product meant to be spread on or sandwiched in a base like bread e.g. cheese spread, mayonnaise, and they form a large constituent of the present market. Hence, the present study was carried out to develop a meat spread from spent hen meat, with lowered or no external fat added and to develop a healthier alternative to already existing meat products utilizing undervalued meat with lowered quantities of fat.

## Materials and Methods

### Ethical approval

Permission of Animal Ethics Committee of Indian Veterinary Research Institute was taken for slaughter of experimental birds.

### Location

The study was undertaken at Indian Veterinary Research Institute (IVRI), Izatnagar, Bareilly, India located at 79° 41’E latitude and 28° 36’N longitude. The place has a hot semi-arid climate and situated at 320 m above mean sea level.

### Spent hen meat and other ingredients

Hens required for the experiments were procured from Central Avian Research Institute (CARI), Izatnagar. Spent hens above 52 weeks of age and weighing between 4.5 kg and 5 kg were slaughtered in the experimental abattoir of the Department of Livestock Products Technology under standard conditions. Carcasses were deboned after 24 h of chilling at 4±1°C. All the visible fat, fascia and connective tissue, was trimmed off and meat was minced twice through a 4.5 mm sieve in a meat mincer (Santos, France).

The meat after packaging in colorless low density polyethylene (LDPE) bag was conditioned for about 24 h at 4±1°C in a refrigerator and then maintained at −18±1°C (not more than 2 days later). Before product preparation, the meat was thawed at 4±1°C for 12 h. The condiment paste of onion, garlic, and ginger in the ratio of 3:2:1 was used. Spice ingredients, procured from the local market were dried at 50±1°C for 4 h in a hot air oven. The ingredients were finely ground and sieved. These were added in fixed proportions shown in Table-1 to give the spice mix.

**Table-1:** Composition of spice mix for meat spread.

Ingredients	% in the mix
Coriander powder (Dhania)	25.0
Cumin seeds (Zeera)	12.0
Dried ginger (Sont)	10.0
Aniseed (Soanf)	10.0
Black pepper (Kali mirch)	10.0
Caraway seed (Ajowan)	5.0
Turmeric (Haldi)	5.0
Capsicum (Mirch powder)	8
Cardamom (Bada elaichi)	5
Cinnamon (Dal chini)	5
Cloves (Laung)	3
Nutmeg (Jaiphal)	1
Mace (Jaipatri)	1
Total	100

### Processing of meat spread

**Figure-1:**
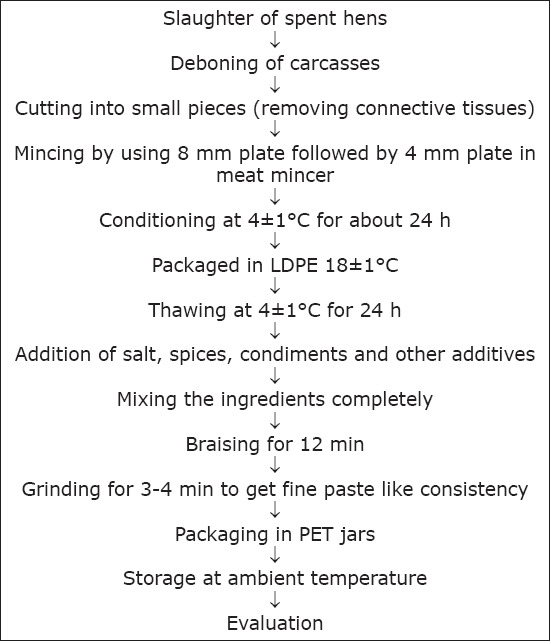
Processing protocol for the development of meat spread.

The following standardized formulation and procedure was used for the processing of meat spread from different groups (Table-2 and Figure-1). The minced meat and ingredients were weighed and mixed properly to form a batter in which all the ingredients are completely mixed. This was then braised at a temperature of 85±2°C. After cooking, the material was grinded in a grinder for 3-4 min to get a fine paste like consistency. Immediately after grinding the products were packaged in food grade PET jars and stored under refrigeration temperature 4±1°C (Godrej Cold Gold, India). The product was compared for its various physicochemical, microbiological, and sensory quality attributes.

**Table-2:** Standardized formulation for the processing of meat spread.

Constituents	Composition (%)
Spent hen meat	48.3
Salt	2.23
Spice mix	1.47
Skimmed milk powder	1.86
Condiments	5.95
Corn starch	2.97
Water	37.17
Total	100

### Analytical procedures

The pH of the meat spread was determined by immersing combined glass electrode of a digital pH meter (Model CP 901, Century Instruments Ltd, Chandigarh, India) to the homogenate. The moisture, protein and fat estimation was done by oven drying, Kjeldahl nitrogen estimation and Soxhlet extraction with petroleum ether [11]. The calorific value of the sample was calculated using bomb calorimeter. The thiobarbituric acid reactive substances (TBARS) value was determined by distillation method of Tarladgis *et al*. [12]. Pink colored distillate was developed using 2-thiobarbituric acid (TBA) mixed in glacial acetic acid. The absorbance was recorded at 538 nm using a spectrophotometer (Scanning mini SPEC, model SL 177, Elico Ltd, Hyderabad) and multiplied with a factor of 7.8. The TBARS value was expressed as mg malondialdehyde/kg of sample [13].

### Microbiological quality

The microbiological evaluation of meat spread was determined as per standard procedures described by APHA [14]. The culturing media was procured from Hi-Media Laboratories (P) Ltd, Mumbai, India. The preparation of samples and serial dilution were done in a horizontal laminar flow unit (Model YSI-188, Yarco Sales (P) Ltd., New Delhi, India) using sterile peptone water as a diluent and following all possible aseptic precautions. The microbial load was expressed as log_10_ cfu/g after number of colonies was multiplied with reciprocal of the dilution. Although plates showing colonies ranging from 30 to 300 were set as the limit of detection for microbiological methods, plates showing below this limit were also recorded to interpret the difference in counts between groups and days. Plate count agar (M091) was used to enumerate total plate count (TPC). Incubation was done at 37±1°C for 48 h and plates showing 30-300 colonies were counted. Coliform count was done on violet red bile agar (M 049A). The plates were incubated at 37±1°C for 48 h. The number of red purple/pink colonies were counted. The yeast and mold count was done on sterile cooled potato dextrose agar (M 096) medium acidified with 10% sterilized tartaric acid solution (1 ml/100 ml of media). The plates were incubated at 25°C for 7 days. All colored colonies that appeared on the plates were counted.

### Sensory evaluation

The sensory evaluation of the products was done by experienced panel consisting of scientists of Division of LPT, IVRI, Izatnagar, India, who judged the samples as per the guidelines of American Meat Science Association, 1995. An 8-point descriptive scale following standard sensory evaluation method was used. The products were evaluated for the appearance, flavor, spread ability, texture, after taste, adhesive ability, and overall acceptability using where 8 is excellent and l is extremely poor [15].

### Statistical analysis

The results of the experiments generated for different quality parameters were evaluated, amassed and analyzed using SPSS (version 20.0) The data were subjected to ANOVA and least significant difference [16] for comparing the means at 5% degree of significance.

## Results and Discussion

The ready-to-eat meat spread contained 58.75±0.32% moisture, 9.12±0.44% crude protein, 11.19±0.16% ether extract, and 2.35±0.17% total ash. The proximate characteristics of the meat spread developed were in agreement with the reports of Das *et al*. [17]. The pH of the meat spread ranged from 6.43±0.01 to 6.21±0.01 in the entire study period and its value showed a descending progression. The decrease in pH might be due to the growth of Lactic acid bacteria in the advanced stages of the storage. TBA values (mg malondialdehyde/kg) increased from 0.157±0.02 to 0.374±0.01 from 0 day to 21^st^ day storage and significant differences (p<0.05) were recorded among the storage periods. Mean TBARS values increased during storage of meat spread showing lipid oxidation, the finding is also supported by previous studies [18]. The increase in TBA values during the storage period due to oxidative rancidity had been reported by many workers [19,20].

The TPC of the meat spread recorded at various periods were found to differ significantly (p<0.05). There was a significant increase in TVPC from 2.85±0.04 cfu/g at zero day to 4.88±0.01 log_10_ cfu/g on 21^st^ day of storage. During the storage period of 15 days, gradual increase in the aerobic and psychrophilic counts were also reported by Bhoyar *et al*. [21] and Mandal *et al*. [22]. The absence of the preservatives might be responsible for the upsurge in the microbiological count with the passage of storage time. As per Cremer and Chipley, the aerobic bacterial count of log 5.33 and psychrophilic count of log 4.6 is considered indicative of unacceptability for cooked meat products [23]. Coliforms and yeast and molds were not detected throughout the storage period of 21 days, which might be due to due to hygienic processing practices and cooking of product to an internal temperature of 80°C. Similar results were reported in which no coliforms were detected during storage of chicken sausages and cooked chicken rolls at refrigeration temperature by Pal *et al.*, [24] and Sachdev and Gopal [25], respectively. On the basis of results obtained by various parameters, it could be said that the meat spread would remain safely shelf stable up to 21 days at refrigeration temperature (Table-3).

**Table-3:** Physicochemical and microbiological characteristics of meat spread at refrigeration temperature (mean±SE)*.

Treatments	Refrigerated storage (days)			

	0 days	7 days	14 days	21 days
pH	6.43±0.01^a^	6.33±0.01^b^	6.22±0.01^c^	6.21±0.01^c^
Water activity	0.975±0.001^a^	0.964±0.001^b^	0.956±0.001^b^	0.946±0.001^c^
TBA (mg malondialdehyde/kg)	0.157±0.02^a^	0.232±0.01^b^	0.313±0.02^c^	0.374±0.01^d^
TPC (log_10_ cfu/g)	2.85±0.04^a^	2.98±0.02^b^	3.79±0.01^c^	4.88±0.01^d^
Psychrophilic count (log_10_ cfu/g)	ND	1.64±0.09^a^	2.39±0.02^b^	3.45±0.01^c^
Coliform count (log_10_ cfu/g)	ND	ND	ND	ND

n=6 for each treatment,

* means with different superscript (row-wise) differ significantly (p<0.05), ND: Not detected, SE: Standard error, TBA: Thiobarbituric acid, TPC: Total plate count

Sensory evaluation revealed significant differences along the storage period in respect of appearance, flavor, spread ability, texture, after taste, adhesive ability, and overall acceptability on 8 point hedonic scale [15]. The significant decrease in these parameters along the storage period corresponded to the increase in TBA values of the meat product. Meat products show a decrease in flavor scores with storage such as in meat pickle by Das *et al.*, [17]. Sharma *et al.*, [26] also reported the decrease in flavor during storage period in extended restructured mutton chops. The decrease in the overall acceptability scores to the meat spread might be due to the cumulative effect of all other attributes [27] and outcome to the progressive decrease in the mean appearance, flavor, spread ability, texture, after taste, and adhesive ability but the product remained well accepted till the last day of evaluation (Table-4).

**Table-4:** Sensory evaluation of meat spread at refrigeration temperature (mean±SE)*.

Parameter/storage days	Storage days			
	
Parameter	0 day	7^th^ day	14^th^ day	21^st^ day
Appearance	7.26±0.03^a^	7.17±0.03^a^	7.04±0.04^b^	6.70±0.04^d^
Flavor	7.14±0.03^a^	7.07±0.02^a^	6.70±0.03^b^	6.69±0.03^d^
Spread ability	7.17±0.03^a^	6.95±0.02^b^	6.90±0.03^c^	6.58±0.02^c^
Texture	7.09±0.02^a^	6.93±0.03^b^	6.94±0.02^c^	6.78±0.04^c^
After taste	7.12±0.03^a^	6.90±0.03^b^	6.70±0.02^c^	6.85±0.03^d^
Adhesive ability	7.08±0.03^a^	6.86±0.05^b^	6.90±0.03^c^	6.76±0.03^d^
Overall acceptability	7.13±0.03^a^	6.89±0.04^b^	6.89±0.03^c^	6.65±0.04^d^

n=6 for each treatment,

* means with different superscript (row-wise) differ significantly (p<0.05), SE: Standard error

## Conclusion

The meat from spent hens can be efficiently utilized to formulate value added ready-to-eat meat spread with high sensory acceptance and high nutritive value, which remains safe for the consumption up to 21 days at refrigeration temperature.

## Authors’ Contributions

The present study was a part of AK’s original research work during his M.V.Sc. thesis program. SKM, ARS, GK and ST conceptualized the aim of the study, designed, planned and supervised the experiment and corrected the manuscript. AK: Collection of samples, execution of the experimental study, collation and analysis of data, interpretation of the results, and drafting of the manuscript. HS, AS, AI and SK helped in analyses, draft, and revision of the manuscript. All authors read and approved the final manuscript.
